# Checklist for managing critical patients' daily awakening

**DOI:** 10.5935/0103-507X.20190057

**Published:** 2019

**Authors:** Juliana Tavares de Lima, Renata Flávia Abreu da Silva, Allan Peixoto de Assis, Alexandre Silva

**Affiliations:** 1 Universidade Federal do Estado do Rio de Janeiro - Rio de Janeiro (RJ), Brasil.; 2 Universidade Federal do Rio de Janeiro, Campus Macaé - Rio de Janeiro (RJ), Brasil.

**Keywords:** Critical care, Conscious sedation, Protocols, Surveys and questionnaires

## Abstract

**Objective:**

To validate the "Checklist for Managing Critical Patients' Daily Awakening" instrument.

**Methods:**

This was a descriptive study that used a quantitative approach for content validation using the Delphi method to obtain the consensus of experts who evaluated the instrument using a Likert scale. The validity index of each item of the instrument was calculated, with a minimum consensus parameter above 0.78.

**Results:**

Three Delphi rounds were required, starting with 29 experts and ending with 15 experts who were invited in person and via e-mail to participate in the study. Of the 15 items in the instrument, 13 had a content validity index > 0.78. The instrument maintained its attributes, and six items were reformulated without the need to exclude any of them. The validated items enabled the assessment of and decisions regarding the dimensions related to the level of sedation and agitation, vital signs, ventilatory parameters and pain. The instrument presented psychometric indicators with acceptable content validity.

**Conclusion:**

The instrument proposed in the study exhibited content validity for most of its items and emerges as a practical strategy for the management of the daily interruption of sedation of critical patients.

## INTRODUCTION

There is a certain disparity between the need for sedation and the way sedation is provided. Inadequate sedation may result in pain, anxiety, agitation, unplanned tracheal extubation and catheter withdrawal.^(1,2)^ On the other hand, the use of excessive or prolonged sedation favors prolonged mechanical ventilation (MV), along with delirium, pneumonia, coma, pressure ulcers and longer stays in the intensive care unit (ICU).^(1-5)^

The concept of daily interruption of sedation (DIS), or "daily awakening", aims to assess the need for sedatives and reduce the systemic accumulation of the drug. This strategy is used in approximately 30% of ICUs and can avoid complications caused by excessive sedation in an individualized manner.^(1)^

Daily interruption of sedation is mainly performed in patients under MV to prevent ventilator-associated pneumonia and its harmful effects. Studies have shown that this technique occurs primarily by interrupting the infusion of the drug for a period of time every day until patients become more alert and can achieve earlier weaning from sedation and MV.^(2,3,6,7)^

Other methods with a relevant role in the management of sedation are also described, namely "intermittent sedation", which allows intermittent administrations of the drug, based on the patient's responses, and "goal-directed sedation", which provides the drug at levels that match the clinical needs of the individual. It should be noted, however, that the Clinical Practice Guidelines from 2013 regarding the control of pain, agitation and delirium recommend mild sedation whenever possible.^(8,9)^

Daily interruption of sedation first emerged in 2000 with the publication of a large-impact study, which divided 150 patients into two groups. In the group for which DIS was the intervention, the administration of sedative drugs was interrupted once daily, and patient awakening was assessed. The patients were then resedated with a targeted sedation level of between 2 and 3 on the Ramsay Sedation Scale. The control group received traditional care without awakening and without a sedation level target. This brief interruption showed benefits such as shorter MV time and a shorter length of hospital stay, marking the beginning of a new era in the approach to patients under tracheal intubation.^(10)^

This led to the development of several protocols for DIS, with the goal of achieving similar outcomes in terms of MV time and length of hospital stay. In general, as soon as a patient is identified as a candidate for DIS, the sedation infusions are discontinued until he/she is awake and shows signs of responsiveness or intolerance upon awakening (agitation). If there is clinical intolerance of interruption of sedation, continuous infusion can be restarted, usually using half the dose that was previously being used, and the patient should be monitored to determine the ideal dose.^(11)^

However, although the DIS procedure has been described, it is necessary for health professionals working in the ICU to both perform bedside surveillance and take a more detailed and practical approach to the steps of daily awakening so that the procedure is performed in a safe way and with minimal damage.

In this context, the "Checklist for Managing Critical Patients' Daily Awakening" instrument, which is based on the best scientific evidence on the subject, was developed to support ICU professionals in clinical evaluation of and decision-making regarding the daily awakening of these patients.

To ensure that this instrument covers the content that it aims to assess and the topics necessary for DIS management, the present study aimed to validate the "Checklist for Managing Critical Patients' Daily Awakening" instrument.

## METHODS

A descriptive study with a quantitative approach was conducted from March 2016 to March 2018, registered in the Brazil Platform under CAAE number 62771416.7.0000.5285 and approved under opinion number 1,869,349.

In the validation process for the "Checklist for Managing Critical Patients' Daily Awakening" instrument, the Delphi method was used to obtain expert consensus from physicians and nurses with specialization or residency programs in ICU or high-critical care.

Intentional sampling resulted in the selection by convenience of the experts according to the following criteria, which separated them into two groups for validation: experts who had at least 2 years' clinical experience in critical patient care, and experts from the academic field who worked as professors/researchers and had knowledge on the topics addressed in this study, as supported by articles or book chapters published and classes taught. The experts in Group 1 were contacted by electronic means or by direct contact by the principal investigator, and those in Group 2 were contacted by accessing their curriculum in the Lattes Platform on the website of the *Conselho Nacional de Desenvolvimento Científico e Tecnológico* (CNPq).

The instrument was structured using a digital questionnaire in Google Drive^®^ and made available to the expert via email after his/her agreement to participate in the study. At the end of the questionnaire, there was an open-ended question that allowed the expert to provide his/her opinion/contribution.

After reading the answers to the questionnaires on the checklist and computing the data, the instrument was organized to evaluate the consensus of experts according to the Delphi method, using a 7-point Likert scale ranging from 1 (strongly disagree) to 7 (strongly agree).

Microsoft Excel 2010^®^ software was used for data tabulation and calculation of the content validity index (CVI) of each item of the instrument.

To consider the item validated, items with scores of 6 and 7 were defined as valid; the items marked with scores of 3, 4 and 5 were defined as undetermined; and items scored as 1 and 2 were defined as not validated*.*

The following criteria were considered for defining an acceptable rate of agreement among experts: in the case of five or less experts, all should agree; in the case of six or more experts, the rate of agreement should be greater than 0.78.^(12,13)^ This calculation, which yields the CVI, refers to the "proportion or percentage of judges who are in agreement about certain aspects of the instrument and its items"^(12)^ and recommends a CVI of at least 0.78 for a group of six to ten experts.^(12-14)^

## RESULTS

Forty-two nurses and 12 physicians were invited to participate in the study to be experts. Among these, 29 participants completed the questionnaire. Both the professionals working in the ICU where the instrument was developed and the professionals invited via the Lattes Platform participated in the three rounds used for reaching consensus.

The results correspond to the three rounds used to reach expert consensus. In the first round, there was high agreement among the experts' individual evaluations of the items of the instrument, i.e., among the 15 items in the instrument, 8 achieved expert agreement and did not require any changes.

Among these items, two had a CVI of 0.96, and the other six had a CVI between 0.93 and 0.79. Items 2 and 3 obtained the highest agreement, with only one non-agreement. Item 2 was "Report the sedation (drug) used", and item 3 was "Check for any prescribed analgesic."

The other items were reformulated according to the suggestions, and the experts were invited via email for a second round. After emphasizing the importance and relevance of keeping nonvalidated items in the checklist and with the support of bibliographic references, high agreement was observed in the second round, with the participation of 19 experts who were able to validate three more items with CVIs ranging from 0.89 to 0.100: item 5, "Check for any prescribed antipsychotic medication" (CVI = 0.95); item 9, "In the 12th hour after the interruption, assess the need to resume sedation if clinically indicated" (CVI = 0.89); and item 15, "In case of failure in the 4 previous steps, resume sedation at the dose described in item 2" (CVI = 0.100). Based on the CVI of the three items validated, it was observed that explaining how the literature supports each item aided in expert decision-making and contributed to the validation of the items. Item 15, for example, reached 100% validation, with agreement from all the experts who participated in this round.

The nonvalidated items were again reformulated, and the experts were invited via email for a third round.

In this round, which included 15 experts, two items were validated, with CVI values of 0.80 and 0.100; however, two items with CVI values of 0.46 and 0.66 remained.

In the second round, among the 29 experts who agreed to participate in the study, 19 evaluated the instrument. In the end, 15 experts provided evaluations in the third round.

Among the 15 proposed items, 13 were validated, and two failed to obtain the minimum CVI for validation. The final items included the instrument are presented in table 1 with their respective CVIs.

**Table 1:** Final items after three rounds of evaluation

Item	CVI
1. Identify the patient	0.100
2. Report the sedation (drug) used	0.96
3. Check for any prescribed analgesics	0.96
4. Check for any prescribed benzodiazepines	0.79
5. Check for any prescribed antipsychotic medication	0.95
6. The time to interrupt the sedation will be determined by the unit	0.66
7. Evaluate SAS every hour in the first 6 hours	0.83
8. After the 6^th^ hour, evaluate SAS every hour	0.46
9. At the 12^th^ hour after interruption, assess the need to resume sedation or whether sedation is clinically indicated	0.89
10. Evaluate the need to resume sedation infusion at half the previous dose	0.80
11. If SAS ≥ 5 - 1^st^ step - assess vital signs	0.93
12. If SAS ≥ 5 - 2^nd^ step - assess ventilatory parameters	0.89
13. If SAS ≥ 5 - 3^rd^ step - assess pain using the BPS scale	0.86
14. If SAS ≥ 5 - 4^th^ step - report to the medical team to reassess the drug dose	0.89
15. In case of failure in the 4 previous steps: resume sedation at the dose described in item 2	0.100

CVI - Content validity index; SAS - Sedation-Agitation Scale; BPS - Behavioral Pain Scale.^(24)^

## DISCUSSION

Physicians and nurses who worked in the ICU where the instrument was developed and used were invited to participate in the study as their experience in the unit's daily practice was considered essential for the checklist's adequacy and validation. However, despite using the instrument under consideration in their professional practice, the number of experts who participated with the study was smaller than the number invited via the Lattes Platform.

Recruiting experts to participate in the study came with some difficulties, despite identifying professionals who met the established inclusion criteria. This is because most of them did not answer the email to confirm their participation in the study, and there was also a delay in returning the material sent to them. Situations such as this have been pointed out by other authors and are among the difficulties of validation studies.^(12,15)^

The validated items reached values ranging from 0.79 to 0.100, with some reaching maximum consensus. However, this level of validation happened after the researchers explained how the individual items were supported by the literature.

In the validation studies found in the literature, with regard to the number of experts as well as agreement, three studies have worked with fewer than six experts.^(16-18)^ However, all of those studies used content validity and were able to obtain agreement and thus validate their instruments.

Another aspect to consider concerns the number of items in the instruments studied. The most discrepant number was found in a study that evaluated 157 items of the adopted instrument. The initial model consisted of 207 items, but a common observation by the group of judges regarding the large size of the instrument resulted in the exclusion of 50 questions that were considered irrelevant or nonrepresentative.^(16)^

It is important that the instrument developed facilitates clinical practice, but to do so, it is necessary to take into account the population to whom the instrument will be applied or used. It is assumed, therefore, that a short instrument may be easier to apply, which would optimize its use and effectiveness for the population group in question, thus achieving the results desired from its application.^(16)^

Regarding the items discussed in the validation of the checklist for which consensus was not obtained, it should be noted that the literature did not provide information about an ideal time to interrupt the sedation of a critical patient to induce awakening. Only questions about the sleep cycle and physiological vigilance of the patient and the duration of the effect of each drug in question were addressed, as advocated by the *Associação de Medicina Intensiva Brasileira* (AMIB).^(19)^

Taking into consideration that each patient is unique and that, based on their clinical condition, a certain type of sedation is used with a certain dose of continuous infusion for a specific period of time, these factors can influence the decision to interrupt the sedative to cause the patient's awakening. In addition, management factors for each service, shift schedule and various other ICU routines can directly influence the selection of the best time to interrupt sedation. This fact was even cited by one of the experts in the first round of the qualitative evaluation.

For these reasons, two times were suggested for interrupting sedation (6:00 am and 8:00), but no agreement was reached. Thus, it was decided to maintain the last option suggested, which indicates that the time for the interruption of sedation be determined by each unit after discussion between the medical and nursing teams. Thus, in the final instrument, in item 6, a field was provided to report the time selected for interruption of sedation, as established by the unit's protocol.

There was also difficulty reaching agreement for the item defining the evaluation time after the sixth hour. Reassessment of the level of sedation was suggested according to the scale used in the instrument under review, namely, the Sedation-Agitation Scale (SAS), based on the following proposals: after the sixth hour, every 2 hours if necessary, and then every hour if necessary. However, none of the three proposals reached the minimum consensus for validation because the last option obtained the lowest CVI value (0.46), while the first two had higher agreement values.

A study on sedation protocols *versus* daily interruption of sedation systematically reviewed studies that compared a protocol with a mild level of sedation with DIS.^(20)^ In one study, SAS was re-evaluated every 1 - 2 hours in the sedation protocol, while in the DIS protocol, the infusion of sedatives and opioids was maintained in the same way as in the sedation protocol, but the sedatives and analgesics were turned off at 9:00 am.^(21)^ In another study that used the Ramsay scale, sedation was interrupted daily, and awakening was assessed. To maintain a Ramsay score of 3 - 4, the patient was evaluated every 2 - 3 hours.^(22)^

We can therefore observe that sedation protocols and daily interruptions of sedation use various strategies but seem to be equivalent to strategies that aim for milder levels of sedation.

To improve the usability of the checklist and thus facilitate the professional's evaluation regarding the patient's level of sedation/agitation, it was suggested that item 8 comprise the evaluation of the level of sedation and agitation with the SAS after the 6th hour at the same time that vital signs were checked to reduce possible resistance when reassessing the patient.

The study used content evaluation as a method. Despite the lack of consensus on two items, new evaluation rounds with the experts were not performed because other changes in the instrument were no longer possible without the risk of mischaracterizing the instrument.

It is assumed that the fact that the two items were not validated may be associated with the decrease in the number of experts who participated in each round. However, even so, it is believed that this will not affect the use of the instrument. The proposed application of the instrument in everyday practice can contribute to its clinical validation, providing answers and assisting in the evaluation of these items.

Based on the results obtained, it can be concluded that the instrument presented acceptable psychometric indicators of content validity, indicating that it can be used for patients admitted to the ICU. Because the instrument is valid and easy to apply, it can allow better identification of the diseases and the use of appropriate strategies for decision-making in regard to managing critical patients' daily awakening. This 15-item instrument retained its attributes with the reformulation of six items (Table 2) and no exclusions (Table 3).

**Table 2:** Comparison of the items that were modified after the three rounds

Initial items	Final items
1. Indicate the patient's name.	1. Identification of the patient.
6. Interrupt sedation at 8 hours.	6. Interrupt sedation at _______.
8. After the 6^th^ hour, reassess the sedation-agitation level (SAS) if necessary.	8. After the 6^th^ hour, proceed to evaluation of the level of sedation-agitation (SAS) at the same time that vital signs were checked.
9. Define the 12^th^ hour as the last hour to assess the level of sedation-agitation (SAS).	9. At the 12^th^ hour after interruption, assess the need to resume sedation or whether sedation is clinically indicated.
10. Resume sedation after the 12^th^ hour.	10. Evaluate the need to resume sedation infusion at half the previous dose.
15. In case of failure of the 4 steps, resume sedation at the dose previously prescribed.	15. In case of failure in the 4 previous steps: resume sedation at the dose described in item 2.

SAS - Sedation-Agitation Scale. Source: Data extracted from the questionnaire sent to experts via email.

**Table 3:** Checklist for Managing Critical Patients’ Daily Awakening

1. Name: __________________________________ Medical record: __________________________________ Date: _______________________________	
2. Sedation and dose used: _________________________________	
3. Analgesic prescribed: ( ) Yes ( ) No Which one? __________________	
Was it necessary to administer? ( ) Yes ( ) No	
4. Benzodiazepine prescribed: ( ) Yes ( ) No Which one? __________________	
Was it necessary to administer? ( ) Yes ( ) No	
5. Prescribed antipsychotic: ( ) Yes ( ) No Which one? __________________	
Was it necessary to administer? ( ) Yes ( ) No	
6. Sedation stopped at: _________________________________	
7. Assess level of sedation-agitation (SAS) every hour in the first 6 hours:	
1^st^ hour - assess level of sedation-agitation (SAS): _________________	
2^nd^ hour - assess level of sedation-agitation (SAS): _________________	
3^rd^ hour - assess level of sedation-agitation (SAS): _________________	
4^th^ hour - assess level of sedation-agitation (SAS): _________________	
5^th^ hour - assess level of sedation-agitation (SAS): _________________	
6^th^ hour - assess level of sedation-agitation (SAS): _________________	
8. After the 6^th^ hour, proceed to the evaluation of the level of sedation-agitation (SAS) at the same time as the vital signs are checked:	
9. 12^th^ hour – resume sedation if clinically indicated: ( ) Yes ( ) No	
Why? ______________________________________________________________________	
10. Evaluate the need to resume sedation infusion at half the previous dose:	
( ) Yes ( ) No	
11. If SAS ≥ 5	12. If SAS ≥ 5
1^st^ step - assess vital signs:	2^nd^ step - assess ventilatory parameters:
	
BP: _____x_____mmHg MAP: _____	VT: _____________ Pressure peak: ________
HR: _____bpm	Flow: ___________ FiO_2_: _______________
RR: _____irpm	Frequency: _______ PEEP: ______________
SpO_2_: ______%	
	
13. If SAS ≥ 5	14. If SAS ≥ 5
3^rd^ step - assess pain through the BPS scale: __________	4^th^ step - report to the medical team to reassess the drug dose:
	Adjustment required: ( ) Yes ( ) No
	
15. In case of failure of the 4 previous steps:	
Resume sedation at the dose described in item 2:	

SAS - Sedation-Agitation Scale;^(23)^ BPS - Behavioral Pain Scale;^(24)^ BP - blood pressure; MAP - mean arterial pressure; HR - heart rate; RR - respiratory rate; SpO_2_ - blood oxygen saturation; VT - tidal volume; FiO_2_ - fraction of inspired oxygen; PEEP - positive end-expiratory pressure.

Table 4 displays the items validated on the final checklist template, followed by their respective competencies suggested by the multidisciplinary team. Although the feasibility of using the checklist, indicated by the validation of its content after evaluation by the experts, further studies are needed to investigate the effectiveness of the material and the knowledge gained from its use. In addition, the validation of the content of the items that were not included in the third step of the present study and their clinical validation by experts may provide more solid evidence regarding the adequacy of these items for application in everyday clinical practice.

**Table 4:** Script of the “Checklist for Managing Critical Patients’ Daily Awakening” instrument

No.	Action	Evaluation
1	Identify the patient	Made by the nurse
2	Check for sedation and dose used	Made by the nurse
3	Check for any prescribed analgesics	Made by the nurse
4	Check for any prescribed benzodiazepines	Made by the nurse
5	Check for any prescribed antipsychotic medication	Made by the nurse
6	Set the time to stop sedation	Agreed upon between doctors and nurses
7	Assess the sedation-agitation level (SAS) every hour for the first 6 hours.	Made by the nurse with the collaboration of everyone on the team (physician, nurse, physiotherapist and nurse technician)
8	After the 6th hour, evaluate the level of sedation-agitation (SAS) at the same time as vital signs are checked.	Assigned to the nurse technician responsible for the patient under the supervision of the nurse
9	Resume sedation if clinically indicated	Medical decision
10	Resume sedation infusion at half the previous dose.	Medical decision
11	If SAS ≥ 5 1st step - assess vital signs	Made by the nurse
12	If SAS ≥ 5 2nd step - assess ventilatory parameters	Made by the physiotherapist (in his/her absence, evaluation should be made by the nurse or physician)
13	If SAS ≥ 5 3rd step - assess pain using the BPS scale	Made by the nurse
14	If SAS ≥ 5 4th step - reassess the drug dose	Medical decision
15	Failure of the 4 previous steps: resume sedation at the dose described in item 2	Made by the nurse
NOTE: The decision to induce daily awakening in a specific patient should be discussed among physicians, nurses and physiotherapists; this is why *multidisciplinary rounds* are highly recommended. It is important to have the knowledge, participation and commitment of all members of the team. Notes should be taken in the patient's medical records by all professionals, especially in the event of complications. The responsibility for starting, maintaining and safekeeping the checklist falls on the nurse, as does the management of nursing care. Any adverse events related to care should be reported as recommended by the Brazilian Health Regulatory Agency (ANVISA). The checklist should undoubtedly be added to the patient's treatment, respecting the patient’s integrity and safety above all.		

SAS - Sedation-Agitation Scale;^(23)^ BPS - Behavioral Pain Scale.^(24)^

The limitations of this study include the reduced number of study participants and, subsequently, the difficulty of obtaining completed instruments. It is believed that these factors did not significantly affect the results of the study. However, such aspects should be considered in other studies that use a similar methodology. Further studies should be developed to better support evidence-based care in order to positively clinical practice and consequently increase the quality of care provided to critical patients admitted to the ICU via improved outcomes and increased safety.

## CONCLUSION

The instrument was considered valid for the management of daily awakening and suitable for use by professionals working in intensive care units. These characteristics enable its use in the context of care for the target population despite the lack of validation of two items, as they were considered not to hinder the instrument's applicability.
